# Characterizing the spatial distribution of brown marmorated stink bug, *Halyomorpha halys* Stål (Hemiptera: Pentatomidae), populations in peach orchards

**DOI:** 10.1371/journal.pone.0170889

**Published:** 2017-03-31

**Authors:** Noel G. Hahn, Cesar Rodriguez-Saona, George C. Hamilton

**Affiliations:** Department of Entomology, Rutgers University, New Brunswick, New Jersey, United States of America; Universita degli Studi della Basilicata, ITALY

## Abstract

Geospatial analyses were used to investigate the spatial distribution of populations of *Halyomorpha halys*, an important invasive agricultural pest in mid-Atlantic peach orchards. This spatial analysis will improve efficiency by allowing growers and farm managers to predict insect arrangement and target management strategies. Data on the presence of *H*. *halys* were collected from five peach orchards at four farms in New Jersey from 2012–2014 located in different land-use contexts. A point pattern analysis, using Ripley’s K function, was used to describe clustering of *H*. *halys*. In addition, the clustering of damage indicative of *H*. *halys* feeding was described. With low populations early in the growing season, *H*. *halys* did not exhibit signs of clustering in the orchards at most distances. At sites with low populations throughout the season, clustering was not apparent. However, later in the season, high infestation levels led to more evident clustering of *H*. *halys*. Damage, although present throughout the entire orchard, was found at low levels. When looking at trees with greater than 10% fruit damage, damage was shown to cluster in orchards. The Moran’s I statistic showed that spatial autocorrelation of *H*. *halys* was present within the orchards on the August sample dates, in relation to both populations density and levels of damage. Kriging the abundance of *H*. *halys* and the severity of damage to peaches revealed that the estimations of these are generally found in the same region of the orchards. This information on the clustering of *H*. *halys* populations will be useful to help predict presence of insects for use in management or scouting programs.

## Introduction

The spatial arrangement of insect populations in an area could influence actions taken by pest managers, and therefore must be understood. Since insects are mobile organisms governed by the need to feed and reproduce, they often disperse themselves in space in predictable ways. In stores containing food products, Indian meal moth (*Plodia interpunctella* Hübner) has been found concentrated around areas with concentrations of birdseed and pet products [1]. Broadly speaking, insect populations can exhibit either a homogeneous (uniform), random, or clustered arrangement in space [2]. These patterns are affected by the spatial scale of investigation. A uniform pattern is one that is evenly distributed across a landscape. A random pattern of distribution means that individuals are equally likely to occur at any location in the area. A clustered distribution is one in which many individuals are concentrated closely together with large areas containing few or no individuals. This spatially heterogeneous distribution could result from environmental constraints, such as a landscape feature like availability of resources, climate, or soil type. For example, the area of gypsy moth and spruce budworm defoliation has been found to increase or decrease depending on fluctuations in temperature and precipitation [3]. The input of chemicals and physical manipulation on farms results in changes to the landscape. Understanding these changes is important to our understanding of how agricultural pests are spatially distributed.

Spatial heterogeneity generally describes the variability of a distribution of multiple species or a population of organisms across a landscape. Populations of insects might exhibit different distribution patterns in landscapes depending on their context, so spatial heterogeneity is an important factor to take into account when making management choices. These patterns can change throughout the year, as there can be different environmental factors that cause the individuals to move around, such as resource availability or proximity to other fruiting crops. For example, Mediterranian fruit fly, *Ceratitis capitata* Wiedemann, adults were found to aggregate in apricot and peach orchards in late summer, and in apple orchards with ripe fruit in autumn. By November, populations exhibited a random distribution [4].

Spatial clustering can occur at a variety of distances and can manifest at different spatial scales [5]. Scale refers to the spatial extent of ecological processes [6]. This concept can influence how a process is viewed in the field. At shorter distances and smaller spatial scales, individual insects might seem to be evenly distributed. However, when looking at an orchard as a whole or an entire farmscape, clustering or other spatial distributions might become more apparent. For example, there could be a population of insects on only the northern end of an orchard and very few or no individuals on the remainder of the trees. If a farm manager is surveying only the northern portion of this orchard and sees a population of insects on those trees, he or she may apply treatments to the entire orchard when only a portion required treatment. A small extent such as an orchard would be a homogeneous culture of fruit trees, but when observing a larger extent containing the orchard (e.g. a farm), the area would be considered a heterogeneous mix of different crops. Organisms of different sizes (e.g. a mosquito and a bird) will also have varied responses and interactions with these extents. Individual insects might also respond differently to an environment at a smaller or larger scale. Therefore, it is important to examine organisms at multiple scales when conducting research [7,8,9].

The brown marmorated stink bug, *Halyomorpha halys* Stål, is an invasive pest of multiple agricultural and ornamental crops in the United States [10]. The earliest detection of *H*. *halys* was in Allentown, PA in samples collected in 1996, and has since then spread throughout the United States to 42 states, where it is considered a severe agricultural pest in six states and has agricultural pest status in eight states [11,12]. *Halyomorpha halys* has a significant impact on stone fruit, and has, in years experiencing large infestations, caused almost complete crop loss to some growers [13]. When damaged by the insect, the stone fruit becomes unmarketable due to damage to the fruit tissue or deformation of the fruit itself [14]. Recent years of heavy infestation have increased the insecticide and management input for farmers, reducing the effectiveness of established IPM programs [15]. *Halyomorpha halys* has the ability to feed on multiple plants, and with this trait has been able to spread quickly across the landscape. In less than 20 years since its detection, it has established populations throughout the majority of the lower 48 states. Because of the ubiquity of this pest and the threat it poses to our food security, informed and focused tactics would save costs and improve efficacy of management of *H*. *halys*.

Using GIS and exploratory analysis of data can lay the groundwork for further investigations into the underlying mechanisms behind spatial patterns [16]. With more accessible ways to visualize and explain spatially referenced data through software and statistical packages, researchers and managers are becoming more cognizant of the importance of spatial heterogeneity. A Ripley’s K test can be used to determine whether or not clustering occurs in a spatial process. This test has been used to explain the spatial distribution of a wide range of subjects, including tree species, gopher mound locations, and road-kill [17,18,19]. It has been extended to insect populations; caddisflies were found to be clustered along with rocks used as oviposition sites in two stretches of streams at different spatial scales [20]. A Moran’s I can be used to test for spatial autocorrelation of points in an area. Johnson and Worobec (1988) showed that grasshoppers in Alberta are autocorrelated at a geographic scale [21]. In addition to these statistical tests, semivariograms are used to characterize the spatial structure of data, and are closely associated with kriging, an interpolation method commonly used to predict values within a spatial framework. Kriging has been used as a forecasting model of gypsy moth defoliation and estimate densities of Colorado potato beetle for site-specific management [22,23]. In this study, we looked at how clustering of *H*. *halys* populations in peach orchards changed over time, and in several different landscape contexts. Additionally, we examined one orchard in detail by investigating all sampled dates for whether or not damage by *H*. *halys* clustered in tandem with its presence in the field. We hypothesized that low populations of *H*. *halys* early in the summer would have low levels of clustering and their spatial distribution would be randomly distributed or dispersed, likely due to lack of ripe fruit. Later in the season, when fruit has matured and population levels are higher, clustering would occur and these populations would exhibit higher levels of spatial autocorrelation than smaller populations. This should occur regardless of the land use surrounding each site. This understanding of spatial patterns and spatial heterogeneity would benefit the development of not only insecticide applications, but also sampling methods and the predictions of insect movement and dispersal. Our specific objectives were to determine the time of season at which clustering of *H*. *halys* will most likely occur, and if populations are spatially autocorrelated in the orchard.

## Materials and methods

### Ethics statement

No endangered or protected species were involved in this study. We obtained permission from private farmers for access and data collection in their orchards.

### Site selection

Five orchards at four farms were selected to be monitored based on region and landscape context. Two of the farms are research and extension centers owned by Rutgers University, the Rutgers Fruit and Ornamental Research and Extension Center (Cream Ridge) in Cream Ridge, New Jersey, and the Rutgers Agricultural Research and Extension Center (RAREC) in Bridgeton, New Jersey. Multiple crops are cultivated and grown at these research stations. The other two farms, one in Hunterdon County, New Jersey (Farm N) and one in Gloucester County, New Jersey (Farm S), are commercial farms that contain multiple peach orchards. Farm N has multiple crops and smaller peach orchards than Farm S, which is primarily peach orchards. Peach orchards at each farm were selected for sampling based on information gathered from extension scouts and farmers about *H*. *halys* infestation levels in earlier years. Infestation levels were, in the past, considered to be moderate to high in the orchards selected for sampling. Orchards were of mixed variety, and the immediate landscape around separate orchards at the same farm was different. The landscape context, categorized broadly into agriculture, barren land, forest, urban, wetlands, and water within 1 km and 5 km around each orchard, was calculated by creating buffer regions around each orchard using the two distance categories, then quantifying the amount of area distinguished as each land-use category. Land-use shapefiles were from the 2012 New Jersey Department of Environmental Protection assessment of land-use land cover [24].

**Fig 1:**
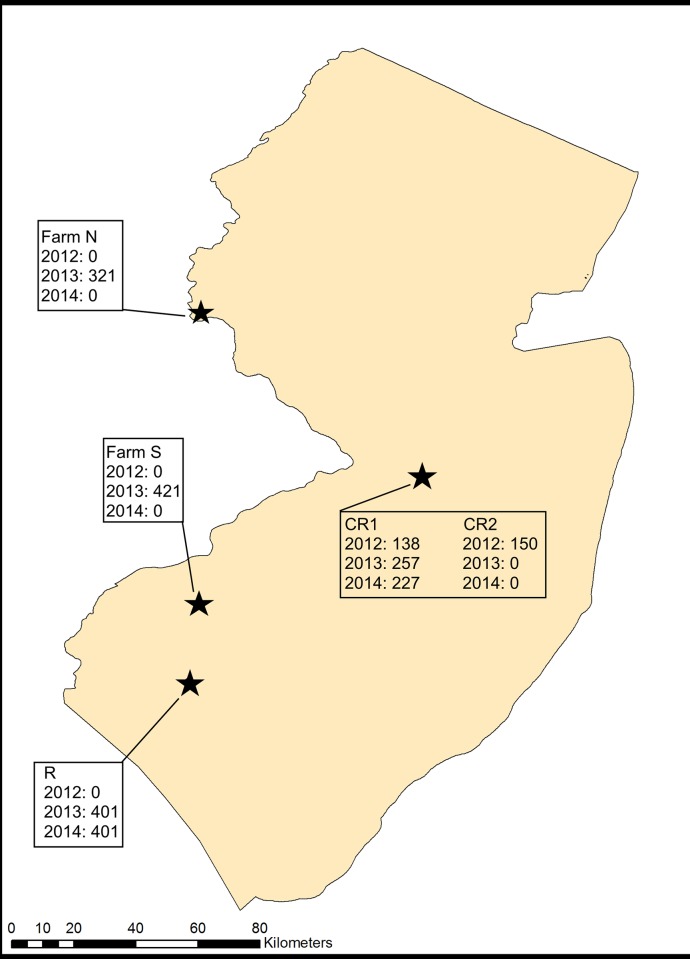
Locations of the surveyed sites and the number of sampled trees in orchards from 2012–2014.

Two orchards were selected for sampling at Cream Ridge [CR1 (1 hectare, Lat: 40.116358, Long: -74.524597), CR2 (0.55 hectare, Lat: 40.117880, Long: -74.522183)], one at RAREC [R1 (1.64 hectares, Lat: 39.516111, Long: -75.200391)], one at Farm N [N1 (2.3 hectares)], and one at Farm S [S1 (4.5 hectares)]. Orchard composition changed year-by-year due to removal or loss of trees or expansion of sampling area, so the number of sampled trees in each orchard each year varied. The orchards at Cream Ridge, CR1 and CR2, are located in central New Jersey, Farm N is located in northwestern New Jersey, and Farm S and the RAREC are located in southeastern New Jersey (Fig 1). Since there were a large number of sampling dates at multiple orchards, two dates per site per year were analyzed for signs of clustering. One date represented low, but increasing populations of *H*. *halys* as the fruit was ripening, typically in June or July. The other represented high populations with orchards containing ripe fruit, typically in late July or through August. Thus, the two sampling dates represented distinct periods in *H*. *halys* population growth early and late in the growing season. Blacklight collections of *H*. *halys* adults was conducted on-site at RAREC and Farm N from 2012–2014 and at Cream Ridge in 2014. Scatterplots of abundance are provided for comparison with orchard-collected data.

### Sampling for *H*. *halys*

Sampling for *H*. *halys* was conducted from 2012 through 2014 in peach orchards across New Jersey. This consisted of weekly samples from May through late August at selected orchards. The specific orchards selected for sampling varied in different years depending on infestation level. At sites in years in which low infestation levels (fewer than 5 *H*. *halys* individuals per week) persisted into early-mid July, sampling for the remainder of the season was aborted. The omitted sites were visited at times in which populations were high at other sites to confirm low or nonexistent populations. Sampling consisted of 1.5 minute visual surveys of trees for *H*. *halys* eggs, nymphs, and adults while circling each tree. All trees in the selected orchards were sampled. In 2012, 138 trees were sampled at CR1 and 150 trees at CR2. In 2013, 257 trees were sampled at CR1, 401 trees at R1, 321 trees at Farm N, and 421 trees at Farm S. In 2014, 227 trees were sampled at CR1 and 401 trees at R1 (Fig 1).

### Sampling for damage

In the CR1 orchard, all sampled trees were visually surveyed for damaged peaches on 2 July 2014 and 12 August 14. Damage sampling consisted of 1.5 minute visual counts of the number of peaches that were superficially damaged. This includes peaches that had resin gumming, catfacing, and obvious puncture wounds. This type of damage is characteristic of piercing-sucking insects, and in these fields, *H*. *halys* was the most prevalent damaging piercing-sucking insect. Damage per tree was quantified as the percent of total fruit counted in that time period that was damaged. Since almost all trees had at least one damaged fruit, a tree was qualified as having damaged fruit if it had 10% or more of its fruit damaged to differentiate between those trees with very few damaged fruit and those that with moderate to severe levels of damage. This was needed to conduct the Ripley’s K analysis because the test cannot account for the magnitude of damage.

### Data analysis

For the purposes of this paper, we define a point as an arbitrary location. A point pattern is a set of points in a region or on a surface. The objective of this analysis is to determine if the point pattern exhibits a pattern over an area or if it is randomly dispersed. A point pattern can be distributed in any of the three distribution patterns stated earlier: random, uniform, or clustered. Exploratory analysis of point patterns, including visualization through maps, extrapolation of point densities, and estimation of spatial dependence among points can lead to further analyses investigating how and why these point patterns were created.

In a scenario investigating an insect population’s spatial distribution, we are looking at a point pattern with a number of events in an area and testing for patterns that do not resemble complete spatial randomness. A method for describing whether a point pattern is clustered in space is the Ripley’s K function, which has been used to describe clustering of *Sirex noctilio* F. in pine plantations and the distribution of wooly apple aphid, *Eriosoma lanigerum* Hausmann, in apple plantings [25,26]. This method, unlike other methods of spatial descriptive statistics, uses a wide range of distances, from the shortest distance between points to the furthest distance between points. This is important because spatial clustering can occur at different spatial scales. The clustering is visualized in plots of the K(t) function. Our null hypothesis of a point pattern of an insect population in a field is one of complete spatial randomness. Any deviation from this at specific distances indicates clustering of populations at those distance scales. Deviations above a null confidence envelope are considered clustered, while deviations below the null confidence envelope indicate a more dispersed pattern.

Point process data were mapped from each sample date at each site to visually display the pattern in ArcMap [27]. The Ripley’s K function is defined as

$$K\left(t\right)=\;\lambda^{-1}E\left[N\right]$$

where *λ* is the density of points and N is the number of events within a certain distance from a randomly selected event [28]. The Ripley’s K function was used to explain the spatial arrangement of point patterns, which in this case is the locations of *H*. *halys* within the sampled peach orchard. The K function does not take into account the magnitude of the number of individuals on each tree, but only the presence of individuals. Ripley’s K plots were created and displayed using the *Kest* function in the *spatstat* package in R [29]. Confidence intervals were created using the *Kest* and envelope functions. The envelope command creates confidence envelopes by using Monte Carlo simulations testing the null hypothesis of complete spatial randomness.

Tobler’s law states that “everything is related to everything else, but near things are more related than distant things” [30]. Samples taken from different locations might not be independent. Spatial autocorrelation measures the similarity of a set of spatially distributed points and their associated values [31,32]. In comparison to randomly associated points, positively autocorrelated points are more similar and occur near one another while negatively autocorrelated points are less similar than expected. The Moran’s I statistic has been used in ecological analyses to measure spatial autocorrelation.

A Moran’s I was used to test for spatial autocorrelation of the points in the field. This test explains to what degree the occurrence of an event in space is likely to affect the occurrence of an event in a neighboring or nearby space. In order to assess spatial autocorrelation, the observer needs to determine a distance measure that defines what is meant by two observations being close to one another. These distances are placed into a weights matrix that includes relationships between all points. So, if there are n sample points, this weights matrix will be n × n. The Moran’s I statistic uses this weights matrix, where the formula for Moran’s I is

$$I=\frac{N\sum_{i=1}^{n}\sum_{j=1}^{n}w_{ij}\left(x_{i}-\bar{x}\right)\left(x_{j}-\bar{x}\right)}{(\sum_{i=1}^{n}\sum_{j=1}^{n}w_{ij})\sum_{i=1}^{n}{\left(x_{i}-\bar{x}\right)}^{2}}$$

where N is the number of observations, x is the variable of interest, $\bar{x}$ is the mean of the variable, x_i_ and x_j_ are the variable values at a locations *i* and *j*, and w_ij_ is a weight index of the location of *i* relative to *j* [33]. This statistic varies on a scale of -1 to +1, with -1 indicating a high negative spatial autocorrelation, or a highly dispersed pattern, 0 indicating no spatial autocorrelation, or a random pattern, and +1 indicating high positive spatial autocorrelation, or a clustered pattern. A Z-score and corresponding p-value are calculated which indicate whether or not we can reject the null hypothesis that the point feature values are randomly distributed across the study area. The Z-score is calculated with the following formula:

$$Z=\frac{I-E\left[I\right]}{\sqrt{V\left[I\right]}}$$

Where E[I] = -1/(n-1) and V[I] = E[I^2^]-E[I]^2^. Analysis was conducted in R using the moran.test of the package ‘spdep’.

A semivariogram can also be used to measure spatial autocorrelation in observations at sampled locations, model the relationships among sample locations, and is involved in estimating values at unsampled locations. Semivariance is a statistic defined as:

$$\gamma \left(h\right)=\frac{1}{2n\left(h\right)}\underset{\left(i,j\right):h_{ij}=h}{\sum }{\left(x_{i}-x_{j}\right)}^{2}$$

Where *γ*(*h*) is the semivariance for distance h, n(h) is the total number of pairs at distance h, and h_ij_ is the distance between locations i and j. The variogram is a plot of the semivariance against distance between pairs of points. The values on the plot increase until it levels off because observations that are closer together should be more similar than points that are separated by a large distance. The value at which the points level off is called the “sill” and the distance to this value on the x-axis is called the “range”. This gives us an idea of the general range of spatial dependence, which is fitted to the plot then used to model the dependence for an interpolation method such as kriging. Semivariograms were created using the “variogram” and “fit.variogram” commands in the ‘gstat’ package in R [29].

Kriging is an interpolation method that is based on the surrounding measured values and the statistical relationships among the measured points. It utilizes a semivariogram model to estimate the value of points at unsampled locations. The distance between points and the variation between points within a specified radius is used to estimate these values. The general formula for a kriging estimator is as follows:

$$Z\left(u\right)-m\left(u\right)=\overset{n\left(u\right)}{\underset{\alpha =1}{\sum }}\lambda_{a}\left[Z\left(u_{a}\right)-m\left(u_{a}\right)\right]$$

where u and u_a_ are location vectors for the estimation point and one of the neighboring data points, n(u) is the number of data points in the local neighborhood used for estimation, m(u) and m(u_a_) are estimation values of Z(u) and Z(u_a_), and λ_a_(u) is the kriging weights. The main goal is to determine weights, λ_a_, that minimize the variance of the estimator. Ordinary kriging was used in this analysis, in which the mean is assumed to be constant in the local neighborhood of each estimation point. Interpolation was conducted to estimate the abundance of *H*. *halys* and the severity of fruit damage in peach orchards at CR1, Farm S, and R1. At each site, assessments for abundance of *H*. *halys* and fruit damage were done on the same date. At CR1, kriging analysis was conducted for sample dates 7 July 2014 and 12 August 2014, at Farm S for 12 August 2013, and R1 for 21 August 2013. Data collection was the same used for the clustering and spatial autocorrelation analysis. This analysis was conducted in R using the “ksline” command of the ‘geoR’ package and the “krige” command of the ‘gstat’ package [29].

## Results

The landscape within 1 km around each orchard varied among the sites. Agriculture was predominant around CR1, CR2, and R1, while forest surrounded the majority of Farm N (S1 Fig). The sites CR and R1 are within highly productive agricultural regions in New Jersey, whereas Farm N is closer to more forested, mountainous areas. The agriculture surrounding these orchards consisted of a mixture of tree fruit orchards, ornamental farms, soybeans, and Christmas tree farms. Plantings at Cream Ridge were comprised of predominantly apricots, apples, and peaches with small amounts of raspberry, strawberry and squash. Farm S was surrounded by a relatively equal mix of agriculture, forest, and urban areas. Within 5 km of each orchard, the patterns of land use remained similar to that within 1 km. However, around CR1 and CR2, agricultural land use dropped from 72% to 46% of the total land use (S1 Fig).

**Fig 2:**
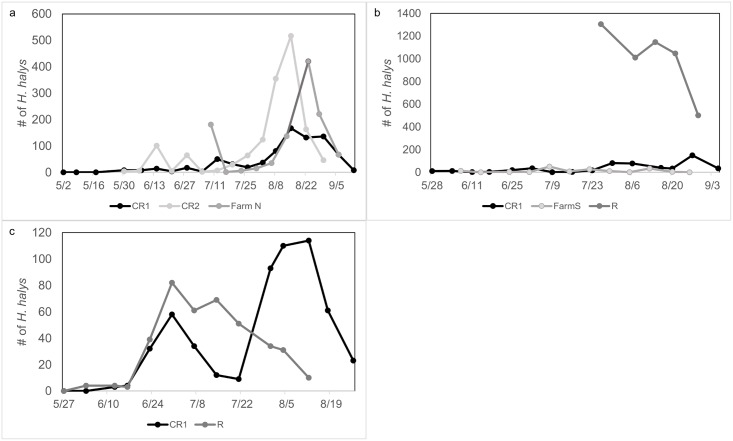
Total number of *H*. *halys* (nymphs and adults) at each site found through visual sampling. (a) 2012, (b) 2013, and (c) 2014.

**Fig 3:**
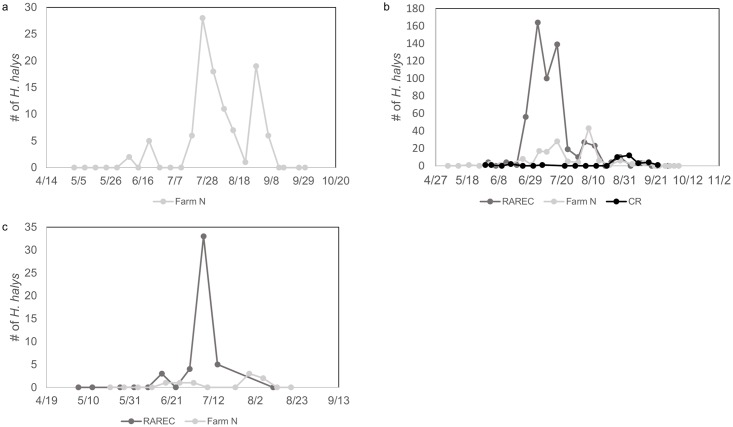
Total number of *H*. *halys* adults found in blacklight traps at sites. (a) Farm N in 2012, (b) RAREC and Cream Ridge in 2013, and (c) RAREC in 2014.

Population densities of *H*. *halys* were higher based on visual sampling towards the end of July and through August (Fig 2). Populations were relatively low in the month of June and early July, with few individuals found on the sampled trees (Fig 2). Blacklight trap captures at Farm N in 2012 showed two strong peaks of *H*. *halys* adults in late July and late August (Fig 3A). Trap captures at RAREC in 2013 indicate high catches from mid-June through July, with lower but still notable populations during sampling (Fig 3B). Few individuals were found in the Cream Ridge blacklight trap at the end of August, differing from the numbers found through our visual sampling (Fig 3B). In 2014, *H*. *halys* were found in high numbers in both the blacklight trap (Fig 3C) and physical sampling (Fig 2C) in late June through August.

**Fig 4:**
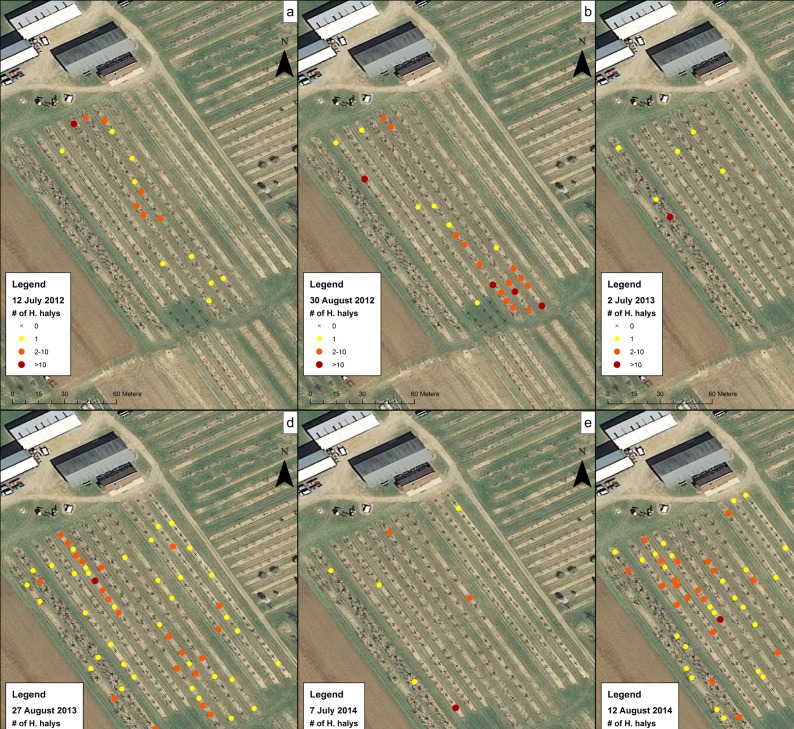
Abundance of *H*. *halys* in the CR1 peach orchard on (a) 12 July 2012, (b) 30 August 2012, (c) 2 July 2013, (d) 27 August 2013, (e) 7 July 2014, and (f) 12 August 2014.

**Fig 5:**
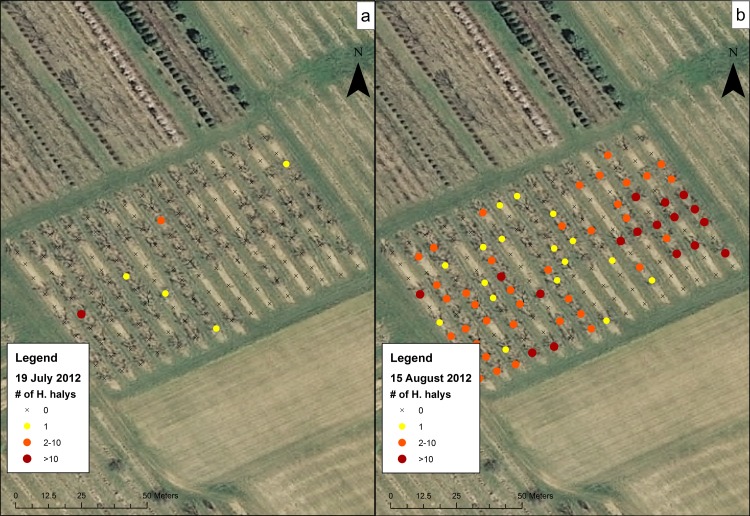
Abundance of *H*. *halys* in the CR2 peach orchard on (a) 19 July 2012 and (b) 15 August 2012.

**Fig 6:**
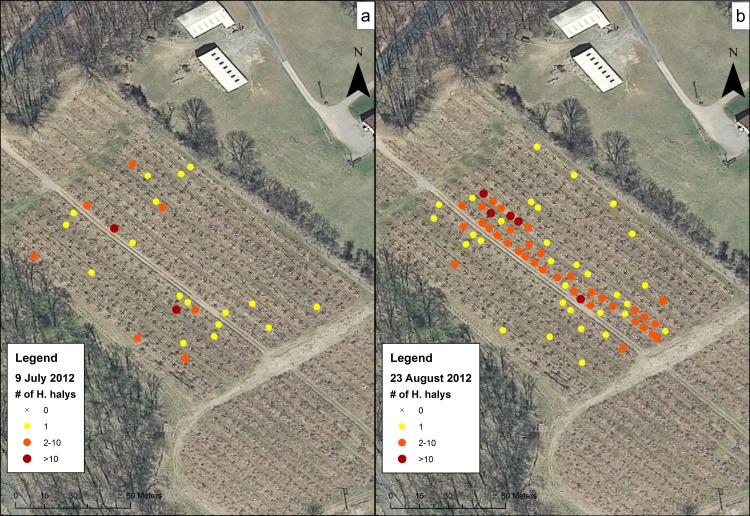
Abundance of *H*. *halys* in the Farm N peach orchard on (a) 9 July 2012 and (b) 13 August 2012.

**Fig 7:**
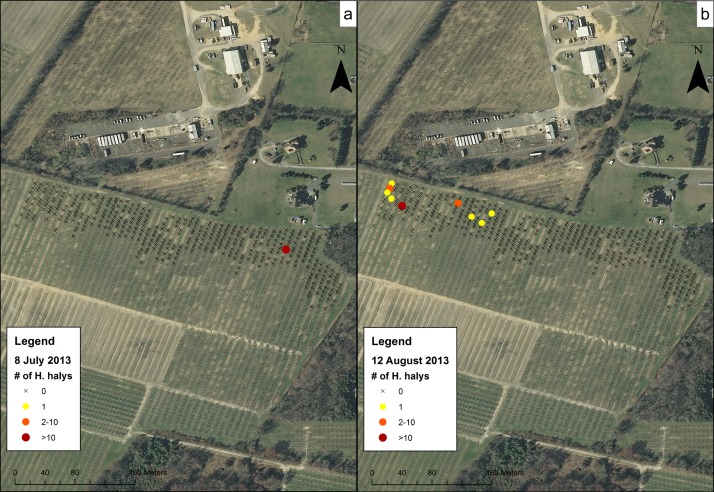
Abundance of *H*. *halys* in the Farm S peach orchard on (a) 8 July 2013 and (b) 12 August 2013.

**Fig 8:**
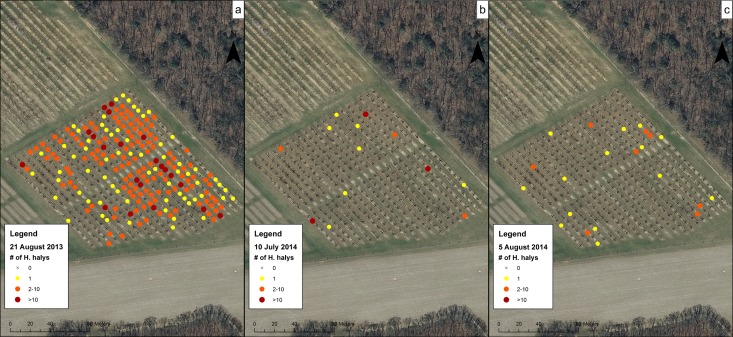
Abundance of *H*. *halys* in the R1 peach orchard on (a) 21 August 2013, (b) 10 July 2014, and (c) 5 August 2014.

Data for the sampling dates chosen at the orchards were mapped to show the presence of *H*. *halys* on trees and to visualize their distribution. These maps showed a representation of high and low levels of clustering of *H*. *halys* across different sampling dates and sites. At CR1 in 2012 (Fig 4a and 4b), although populations were low, *H*. *halys* were found occasionally on the July 12 sampling date and at the southern section of the orchard on the August 30 sampling date. In 2013 and 2014, individuals were found along some of the rows in the middle of the orchard and not the southern-most trees (Fig 4c–4f). Within CR2 on 15 August 2012 (Fig 5), individuals were found throughout the field on most trees. In Farm N (Fig 6), individuals began the season clustered towards the northern part of the orchard, but moved to the middle rows of the orchard later in the season. The number of trees containing individuals in Farm S (Fig 7) was low, and clustering was not apparent. In R1 (Fig 8), individuals were concentrated in rows towards the northeastern half of the orchard. Although these maps are helpful to make general decisions about whether or not populations are clustering, it is difficult to tell at what scale or distance they are exhibiting this behavior.

**Fig 9:**
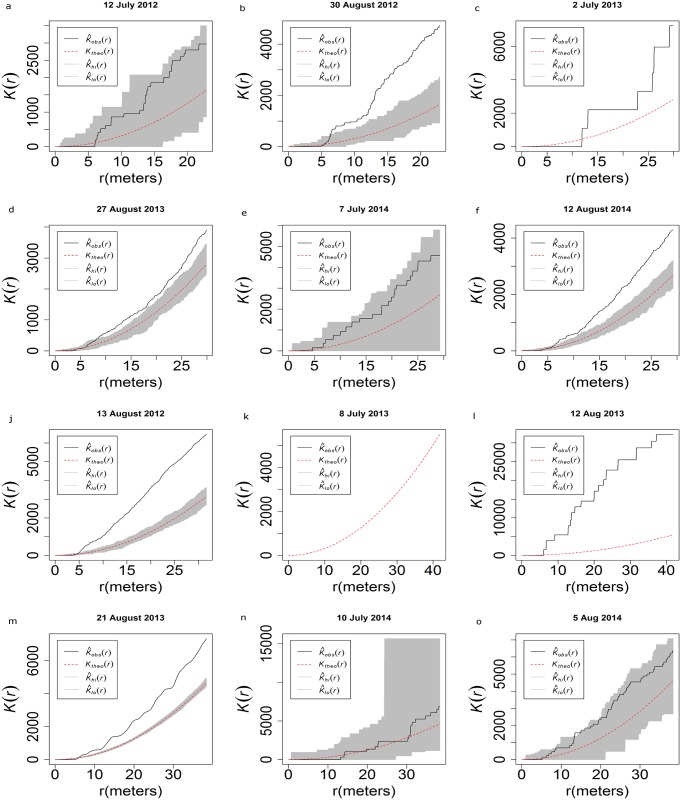
Ripley’s K analysis of the presence of *H*. *halys* at the sampled peach orchards (a) CR1 12 July 2012 (b) CR1 30 August 2012 (c) CR1 2 July 2013 (d) CR1 27 August 2013 (e) CR1 7 July 2014 (f) CR1 12 August 2014 (g) CR2 19 July 2012 (h) CR2 15 August 2012 (i) Farm N 9 July 2012 (j) Farm N 13 August 2012 (k) Farm S 8 July 2013 (l) Farm S 12 August 2013 (m) R 21 August 2013 (n) R 10 July 2014 (o) R 5 August 2014.

The Ripley’s K analysis allows us to graphically examine the distances at which clustering occurs. The red line in the graphs of the Ripley’s K analysis represent a theoretical completely spatially random pattern, and the grey area around it is a 95% confidence envelope of complete spatial randomness. The black line represents the collected data, and shows the degree at which the point process is clustering at different distance classes. Populations in CR1 on 12 July 2012 (Fig 9a) show no clustering, but on 30 August 2012 show clustering to be occurring when points are further than 5 m away (Fig 9b). Subsequent years appear to be the same, with clustering of individuals occurring in August (Fig 9c–9f). CR2, however, shows clustering of populations throughout the season in 2012, although clustering was more pronounced on 23 August 2012 than on 19 July 2012 (Fig 9g and 9h). At Farm N and Farm S (Fig 9i–9l), individuals do not appear to be clustered on 9 July 2012 but clustered on 23 August 2012. At R1 (Fig 9m–9o), clustering occurred in the first year of sampling (2013) but did not appear to cluster in 2014.

The Moran’s I statistics showed that the level of spatial autocorrelation within orchards was low on almost all sample dates except for 23 August 2012 at Farm N and 21 August 2013 at R1. However, at most of the sites in sampled years, there was a significant trend for the Moran’s I statistic in August to be closer to the value of 1 than the value in July (Table 1), showing that the distribution deviates from the expectation of randomly distributed and correlated points.

**Table 1:** Moran’s I statistic for the measurement of spatial autocorrelation in sampled orchards.

Site	Date of sampling	Moran’s I statistic	P-value
CR1	7/12/12	-0.0137	0.5449
	8/30/12	0.0732	0.2057
	7/2/13	-0.0063	0.5666
	8/27/13	0.1891	0.0049*
	7/7/14	-0.0075	0.5740
	8/12/14	0.0637	0.1098
CR2	7/19/12	-0.0127	0.6148
	8/15/12	0.0287	0.3636
Farm N	7/9/12	-0.0117	0.5657
	8/23/12	0.5727	< 0.0001*
Farm S	7/8/13	-2.3809e-3	0.5
	8/12/13	-0.0015	0.4898
R	8/21/13	0.3331	< 0.0001*
	7/10/14	-0.0129	0.583
	8/5/14	0.0765	0.0909

A positive Moran’s I statistic represents positive spatial autocorrelation, while a negative value indicates negative spatial autocorrelation. Values closer to -1 or 1 indicate stronger measures of autocorrelation. The p-value indicates whether or not the null hypothesis of randomly distributed points can be rejected. An asterisk is used to mark significant P-values.

**Fig 10:**
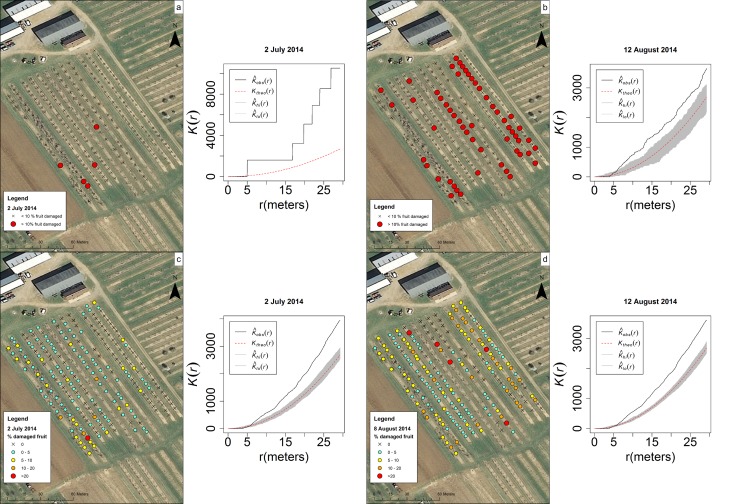
Maps showing the level of fruit damage (greater than 10% damaged fruit) at CR1 on (a) 2 July 2014 and (b) 12 August 2014 and the overall % damaged fruit (c) 2 July 2014 and (d) 12 August 2014 followed by Ripley’s K graphs of clustering.

Damage was assessed in the CR1 peach orchard in 2014 on two separate sampling dates. Maps indicate varying levels of damage throughout the field, and most trees had some damaged fruit (Fig 10). There were more trees with greater than 10% of fruit damaged on the August sampling date than on the July sampling date. Damage clustered on both sampling dates, whether looking at only trees with greater than 10% of fruit damaged (July and August) or the range of damage throughout the field (July), as shown by the level of positive spatial autocorrelation of fruit damage on trees within the orchards (Table 2).

**Table 2:** Moran’s I statistic for the measurement of spatial autocorrelation of fruit damage in CR1 in 2014.

Date of sampling	Level of Damage	Moran’s I Statistic	P-value
7/2/14	% of damage^1^	0.3998	4.472e-7*
	>10% fruit damaged^2^	0.1774	0.0092*
8/12/14	% of damage^1^	0.0974	0.1079
	>10% fruit damaged^2^	0.3529	1.1418e-5*

A positive Moran’s I statistic represents positive spatial autocorrelation, while a negative value indicates negative spatial autocorrelation. Values closer to -1 or 1 indicate stronger measures of autocorrelation. The p-value indicates whether or not the null hypothesis of randomly distributed points can be rejected. An asterisk is used to indicate significant p-values.

^1^ Damaged fruit throughout the field

^2^ Only trees with greater than 10% fruit damaged

**Fig 11:**
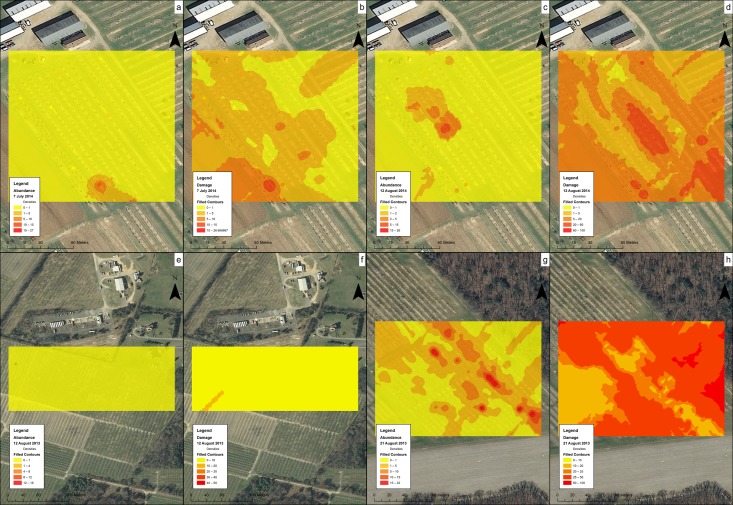
Kriging interpolation of the abundance of *H*. *halys* (a,c,e,g) and the severity of damage (percent of damaged fruit) (b,d,f,h) in peach orchards. (a) CR1, 7 July 2014, *H*. *halys*, (b) CR1, 7 July 2014, damage, (c) CR1, 12 August 2014, *H*. *halys*, (d) CR1, 12 August 2014, damage, (e) Farm S, 12 August 2013, *H*. *halys*, (f) Farm S, 12 August 2013, damage, (g) R1, 21 August 2013, abundance of *H*. *halys*, (h) R1, 21 August 2013, damage. The brighter and whiter the color, the higher the abundance or level of damage.

At CR1 on 7 July 2014, kriging estimated a small population of *H*. *halys* on the southern part of the orchard, and damage assessments showed a larger portion of fruit was damaged in that same part of the field, with some low levels of damage through the remainder of the field (Fig 11a and 11b). On 12 August 2014, only a small population of *H*. *halys* was found in the middle of the orchard (Fig 11c). However, more damage was found at the center of the orchard and the northeastern row of trees (Fig 11d). At Farm S on 12 August 2013, a small population of *H*. *halys* along with low levels of damage was present at the western part of the orchard (Fig 11e and 11f). The orchard at R1 had the highest levels of *H*. *halys* infestation among the sampled sites, and kriging indicated a high level population along the northeastern part of the orchard (Fig 11g). Percent fruit damage was high throughout the orchard, especially towards the southern edge (Fig 11h).

## Discussion

This is the first study to document the distribution of *H*. *halys* in peach orchards. Our results provide strong evidence for clustering that varied over time. There was greater clustering in August at high population levels, and low clustering in July at low population levels. Clustering of instances of damage occurred when levels of damage were high later in the season. Investigating the location and levels of *H*. *halys* populations in orchards enables us to learn about the distribution of the pest in relation to time and space. Since populations of *H*. *halys* varied throughout the sampling season at different sites, we believed that they would exhibit different levels of clustering and spatial autocorrelation throughout this period. Feeding in aggregations has been linked to the tendency of insect populations to outbreak [34]. Our results show that clustering of *H*. *halys* populations occurred mostly on the later sampling dates, when population levels were higher in the orchards. Since this is most prevalent during the August sample dates when fruit is ripe or ripening, it suggests that populations are clustering at times and in areas with suitable resources. In addition, later sampling dates could have had *H*. *halys* immigrants from nearby areas attracted by aggregating individuals already in the orchard. Our kriging analysis of R1 estimated large populations of *H*. *halys* at the northeastern part of the orchard in addition to large levels of damage throughout the area, especially towards the southern section. There is a forested edge past the northeastern part of the orchard, leadings us to believe that many *H*. *halys* migrate from there into the northeastern row. Although there was no indication of high populations of *H*. *halys* in the southern part of the orchard on the day of sampling, the high levels of damage there could be a result of migration to and from an adjacent field of soybeans south of the orchard. A helpful follow-up study investigating the clustering on field edges in relation to the phenology of surrounding crops within the farmscape. A recent study has found that injury to apple due to *H*. *halys* is not uniformly distributed in commercial apple orchards, and injury was generally higher along borders at forested edges [35].

Although trap captures in blacklights were high in late-June through July, it might not necessarily indicate high levels of *H*. *halys* in nearby orchards. Populations could be drawn into the blacklight from other neighboring host plants that are a more suitable food source at that time. The levels of positive spatial autocorrelation between points on August sample dates demonstrate that abundance of *H*. *halys* on one tree is influenced by its abundance on nearby trees. While additional studies are being conducted to determine the abiotic and biotic contributors to this aggregation of *H*. *halys* populations, our analysis presented here shows that there are differences in how populations behave and distribute themselves in the orchard depending on time and space.

There are a variety of factors that contribute to a population of individuals exhibiting a clustered distribution. These could include environmental factors such as availability of food resources, sunlight, refuge, proximity to other infested trees or a number of other biotic or abiotic factors. For example, aphids in an apple planting were shown to cluster within short distances from other heavily infested trees [36]. The stink bugs *Nezara viridula* L. and *E*. *servus* Say have been found to aggregate on a cotton-peanut border when dispersing from peanut to cotton as they switch food resources [37]. Maps of interpolated *N*. *viridula* density show distinct aggregations at borders on sampling dates when the peanut growing season is ending and cotton bolls become a preferred food source. Population levels of *N*. *viridula* have also been shown to be highly influenced by the stage of soybeans in fields [38]. This linkage between resources, landscape and aggregation of individuals suggests that clustering of *H*. *halys* populations could also result from differences in resource availability across a landscape. Studies on *H*. *halys* have begun to examine the prevalence of an edge effect of aggregation in field crop and ornamental settings [39,40]. *Halyomorpha halys* was shown to disperse from corn to adjacent soybean, particularly at the time when soybeans were developing.

Clustering of insect populations can have an impact on the larger context of the landscape and potentially on abiotic factors. For instance, clustering of damage caused by populations of western spruce budworm has been shown to be linked to a decrease in the risk of forest fires in the seven years following infestation [41]. This is thought to be due to the smaller amount of needles in years following defoliation. Our results show clustering of piercing-sucking damage to peaches that is positively spatially autocorrelated. Although other insect species can cause similar damage to peaches, *H*. *halys* was the most common piercing-sucking insect found in the orchard and was likely the cause of most of the damage we observed. In addition, clustering of damage occurred along with clustering of *H*. *halys*. A reduction of the *H*. *halys* population would presumably reduce the amount of damage found in orchards.

Further studies should investigate the distribution of *H*. *halys* in orchards under different population densities. During the current studies, numbers of *H*. *halys* were not as high as in previous years, which might have affected their distribution and aggregation in the field. Less clustering would occur with our model because the model is more robust with larger counts. Additionally, the relative strength of aggregation pheromone at higher levels of population may contribute to the increase in clustering. It would be interesting to investigate and relate the levels and distribution of natural enemies and native pentatomids to *H*. *halys* to see if the potential for predation or competition caused population reductions. Also, we would like to know if these spatial patterns hold when populations are high throughout orchards, i.e. will *H*. *halys* continue to exhibit clustering or show signs of a more uniform or random dispersion in those situations?

Semivariograms, kriging, and the Ripley’s K and Moran’s I statistics have a wide breadth of applications for investigating spatial point processes in nature. From stored product pests, distribution of caddisflies, distribution of grasses and shrubs, determining the spatial structure of sugar maples and even to moose-vehicle collisions these have been powerful tools to display variations in spatiotemporal patterns [42,43,44,45,46]. Findings in our study have implications for management on the farm level. Management strategies might want to consider the location of late-season varieties as well as the location of future plantings of these varieties and the likelihood of attracting aggregations of *H*. *halys*. Since *H*. *halys* clusters and populations are spatially autocorrelated, being aware of their orchards’ structure would help growers predict where the most suitable resources for *H*. *halys* would be located during the growing and harvesting seasons. Scouting in peach orchards should focus on areas with ripening and ripe fruit, then proceed to locations where ripening occurs throughout the orchard and farm. Farm managers should keep in mind that populations of *H*. *halys* are positively spatially autocorrelated in peach orchards on these later dates, and thus individuals are more likely to be closer to other individuals and larger populations. Scouting and management interventions would likely benefit from targeting these areas. Linking clustering of *H*. *halys* to landscape structure, such as food availability and distance to habitat and other resources remains to be seen. Under the range of landscapes in which our sampled orchards are located, clustering was found to different degrees.

## Supporting information

S1 Fig Landscape context around orchards CR1 and CR2 (1km(A) and 5km(B)), Farm N (1km(C) and 5km(D)), Farm S (1km(E) and 5km(F)), and R1 (1km(G) and 5km(H)). (TIF)
