# Supplementary figures and images for: Characterizing the spatial distribution of brown marmorated stink bug, Halyomorpha halys Stål (Hemiptera: Pentatomidae), populations in peach orchards
Source: PLoS One. 2017 Mar 31;12(3):e0170889. doi: 10.1371/journal.pone.0170889 (PMC5376087; doi:10.1371/journal.pone.0170889)

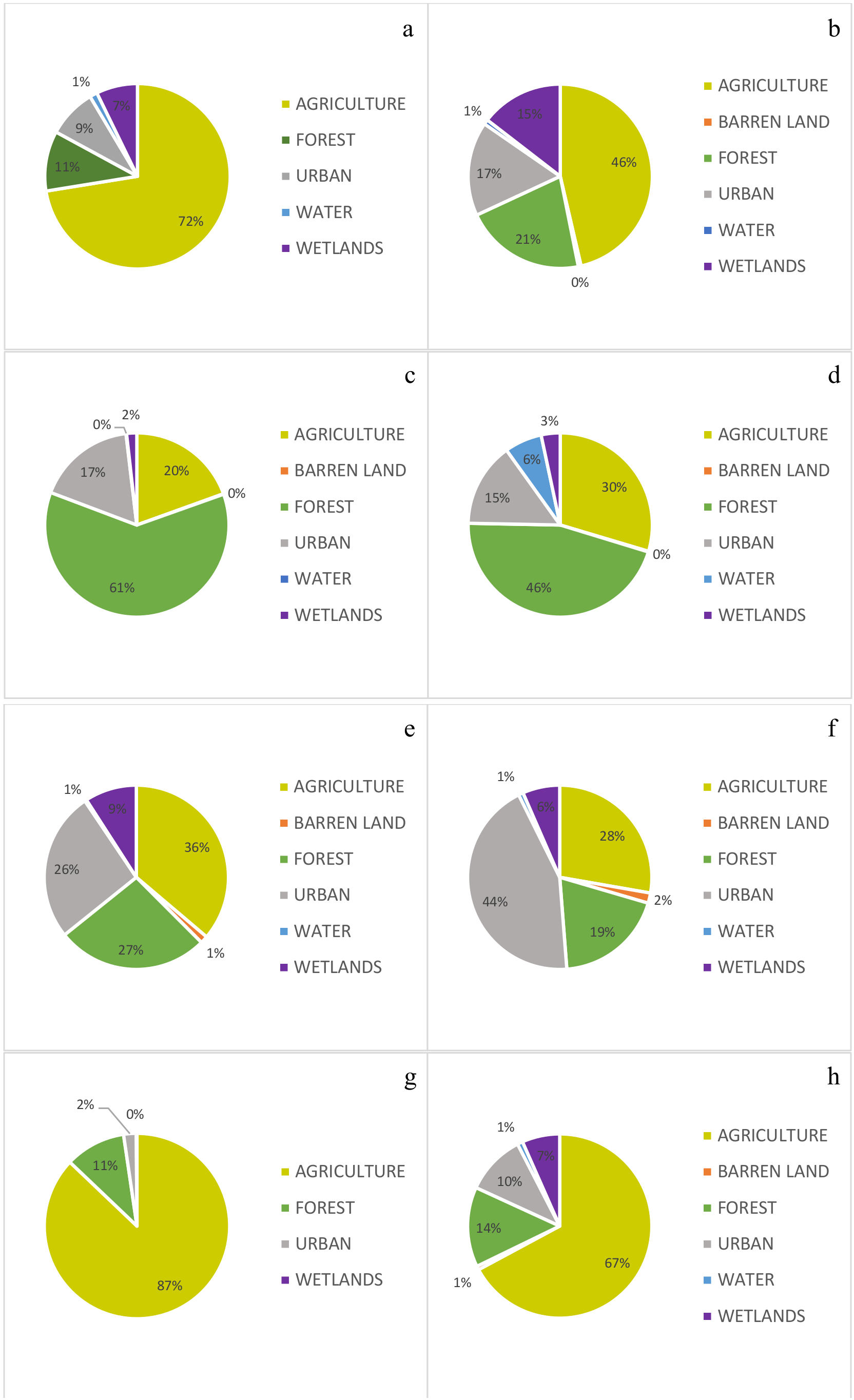

Supplement: S1 Fig — (TIF) [file pone.0170889.s001.tif]
